# Supplementary material for: Modelling Skylarks (Alauda arvensis) to Predict Impacts of Changes in Land Management and Policy: Development and Testing of an Agent-Based Model
Source: PLoS One. 2013 Jun 6;8(6):e65803. doi: 10.1371/journal.pone.0065803 (PMC3675089; doi:10.1371/journal.pone.0065803)
Supplement: Supporting Information S4 — The skylark ODdox as a zipped archive. (ZIP) [file pone.0065803.s004.zip › Skylark_ODdox/landscape_8h.html]

ALMaSS Skylark ODdox: landscape.h File Reference


ALMaSS Skylark ODdox
 2.0


- Main Page
- Related Pages
- Classes
- Files

- File List
- File Members

Classes |
Macros |
Enumerations |
Functions |
Variables

landscape.h File Reference

`#include <vector>`

|  |  |
| --- | --- |
| Classes | |
| class | Landscape |
|  | The landscape class containing all environmental and topographical data. More... |
| class | Point |

|  |  |
| --- | --- |
| Macros | |
| #define | \_CRTDBG\_MAP\_ALLOC |
| #define | TLANDSCAPE\_H |

|  |  |
| --- | --- |
| Enumerations | |
| enum | TTypesOfPesticide {     ttop\_NoPesticide = -1, ttop\_GeneralOrganophosphate = 0, ttop\_EndocrineDisruptor, ttop\_Vinclozolin,     ttop\_ModelinkPesticide, ttop\_ModelinkPesticide21TWA   } |

|  |  |
| --- | --- |
| Functions | |
| int | random (int a\_range) |

|  |  |
| --- | --- |
| Variables | |
| const int | April = 90 |
| const int | August = 212 |
| const int | December = 334 |
| const int | February = 31 |
| Landscape \* | g\_map |
| class Pesticide \* | g\_pest |
| const int | January = 0 |
| const int | July = 181 |
| const int | June = 151 |
| int \* | m\_polymapping |
| const int | March = 59 |
| const int | May = 120 |
| const int | November = 304 |
| const int | October = 273 |
| const int | September = 243 |
| const int | WindDirections [12][100] |

---

## Macro Definition Documentation

#define \_CRTDBG\_MAP\_ALLOC

#define TLANDSCAPE\_H

---

## Enumeration Type Documentation

enum TTypesOfPesticide

**Enumerator:**
:   |  |  |
    | --- | --- |
    | *ttop\_NoPesticide* |  |
    | *ttop\_GeneralOrganophosphate* |  |
    | *ttop\_EndocrineDisruptor* |  |
    | *ttop\_Vinclozolin* |  |
    | *ttop\_ModelinkPesticide* |  |
    | *ttop\_ModelinkPesticide21TWA* |  |

{

ttop\_NoPesticide = -1,

ttop\_GeneralOrganophosphate = 0,

ttop\_EndocrineDisruptor, //1

ttop\_Vinclozolin, //2

ttop\_ModelinkPesticide, // 3

ttop\_ModelinkPesticide21TWA // 4

} TTypesOfPesticide;

---

## Function Documentation

|  |  |  |  |  |  |
| --- | --- | --- | --- | --- | --- |
| int random | ( | int | *a\_range* | ) |  |

References g\_rand\_uni.

Referenced by Landscape::AddBeetleBanks(), UserDefinedFarm::AssignPermanentCrop(), Skylark\_Population\_Manager::Catastrophe(), Skylark\_Base::DailyMortality(), RoadsideVerge::DoDevelopment(), Skylark\_Female::FeedYoung(), Weather::GetSnow(), Landscape::hb\_MarkTheBresenhamWay(), Landscape::hb\_StripingDist(), Skylark\_Population\_Manager::Init(), Landscape::Landscape(), Skylark\_Clutch::OnFarmEvent(), Skylark\_Nestling::OnFarmEvent(), Skylark\_PreFledgeling::OnFarmEvent(), Skylark\_Female::OnFarmEvent(), Skylark\_Male::OnFarmEvent(), SkTerritories::PreProcessLandscape2(), VegElement::RandomVegStartValues(), Landscape::ReadPolys(), Population\_Manager::Shuffle(), Skylark\_Adult::Skylark\_Adult(), Skylark\_Nestling::Skylark\_Nestling(), Skylark\_Male::st\_CaringForYoung(), Skylark\_Nestling::st\_Developing(), Skylark\_Female::st\_Emigrating(), Skylark\_Male::st\_Emigrating(), Skylark\_Male::st\_FindingTerritory(), Skylark\_Female::st\_Floating(), Skylark\_Male::st\_Floating(), Skylark\_Female::st\_Immigrating(), Skylark\_Male::st\_Immigrating(), Landscape::SupplyWindDirection(), Pesticide::Test(), Weather::Tick(), and UnsprayedFieldMargin::UnsprayedFieldMargin().

{

/\* Want to raise exception on this?

if ( a\_range <= 0 )

return 0;

\*/

//int result = (int)(((double) rand() / g\_randmaxp ) \* a\_range);

return (int) (g\_rand\_uni()\*a\_range);

// return result;

}

---

## Variable Documentation

const int April = 90

Referenced by Skylark\_Female::st\_BuildingUpResources(), Skylark\_Female::st\_Emigrating(), and Skylark\_Male::st\_Emigrating().

const int August = 212

const int December = 334

const int February = 31

Referenced by Skylark\_Female::st\_Emigrating(), and Skylark\_Male::st\_Emigrating().

Landscape\* g\_map

class Pesticide\* g\_pest

Referenced by Orchard::DoDevelopment(), OrchardBand::DoDevelopment(), Landscape::Landscape(), Farm::ProductApplication0(), Farm::ProductApplication1(), Landscape::SupplyPesticide(), Landscape::Tick(), and Landscape::~Landscape().

const int January = 0

Referenced by Skylark\_Population\_Manager::DoFirst(), Skylark\_Female::st\_Emigrating(), and Skylark\_Male::st\_Emigrating().

const int July = 181

const int June = 151

Referenced by Skylark\_Female::st\_MakingNest().

int\* m\_polymapping

const int March = 59

Referenced by OrchardGrass::DoDevelopment(), Skylark\_Female::st\_Emigrating(), and Skylark\_Male::st\_Emigrating().

const int May = 120

Referenced by Skylark\_Female::st\_BuildingUpResources(), Skylark\_Female::st\_Emigrating(), and Skylark\_Female::st\_MakingNest().

const int November = 304

const int October = 273

Referenced by OrchardGrass::DoDevelopment(), Skylark\_Female::st\_Flocking(), and Skylark\_Male::st\_Flocking().

const int September = 243

Referenced by Skylark\_Female::st\_Flocking(), and Skylark\_Male::st\_Flocking().

const int WindDirections[12][100]

**Initial value:**

{

{0,0,0,0,0,0,0,0,0,0,0,0,0,0,0,0,0,0,0,0,0,0,0,0,0,0,0,0,0,0,0,0,0,0,0,0,0,0,0,0,0,0,0,0,0,0,0,0,0,0,0,0,0,0,0,0,0,0,0,0,0,0,0,0,0,0,0,0,0,0,0,0,0,0,0,0,0,0,0,0,0,0,0,0,0,0,0,0,0,0,0,0,0,0,0,0,0,0,0,0},

{0,0,0,0,0,0,0,0,0,0,0,0,0,0,0,0,0,0,0,0,0,0,0,0,0,0,0,0,0,0,0,0,0,0,0,0,0,0,0,0,0,0,0,0,0,0,0,0,0,0,0,0,0,0,0,0,0,0,0,0,0,0,0,0,0,0,0,0,0,0,0,0,0,0,0,0,0,0,0,0,0,0,0,0,0,0,0,0,0,0,0,0,0,0,0,0,0,0,0,0},

{0,0,0,0,0,0,0,0,0,0,0,0,0,0,0,0,0,0,0,0,0,0,0,0,0,0,0,0,0,0,0,0,0,0,0,0,0,0,0,0,0,0,0,0,0,0,0,0,0,0,0,0,0,0,0,0,0,0,0,0,0,0,0,0,0,0,0,0,0,0,0,0,0,0,0,0,0,0,0,0,0,0,0,0,0,0,0,0,0,0,0,0,0,0,0,0,0,0,0,0},

{0,0,0,0,0,0,0,0,0,0,0,0,0,0,0,0,0,0,0,0,0,0,0,0,0,0,0,0,0,0,0,0,0,0,0,0,0,0,0,0,0,0,0,0,0,0,0,0,0,0,0,0,0,0,0,0,0,0,0,0,0,0,0,0,0,0,0,0,0,0,0,0,0,0,0,0,0,0,0,0,0,0,0,0,0,0,0,0,0,0,0,0,0,0,0,0,0,0,0,0},

{0,0,0,0,0,0,0,0,0,0,0,0,0,0,0,0,0,0,0,0,0,0,0,0,0,0,0,0,0,0,0,0,0,0,0,0,0,0,0,0,0,0,0,0,0,0,0,0,0,0,0,0,0,0,0,0,0,0,0,0,0,0,0,0,0,0,0,0,0,0,0,0,0,0,0,0,0,0,0,0,0,0,0,0,0,0,0,0,0,0,0,0,0,0,0,0,0,0,0,0},

{0,0,0,0,0,0,0,0,0,0,0,0,0,0,0,0,0,0,0,0,0,0,0,0,0,0,0,0,0,0,0,0,0,0,0,0,0,0,0,0,0,0,0,0,0,0,0,0,0,0,0,0,0,0,0,0,0,0,0,0,0,0,0,0,0,0,0,0,0,0,0,0,0,0,0,0,0,0,0,0,0,0,0,0,0,0,0,0,0,0,0,0,0,0,0,0,0,0,0,0},

{0,0,0,0,0,0,0,0,0,0,0,0,0,0,0,0,0,0,0,0,0,0,0,0,0,0,0,0,0,0,0,0,0,0,0,0,0,0,0,0,0,0,0,0,0,0,0,0,0,0,0,0,0,0,0,0,0,0,0,0,0,0,0,0,0,0,0,0,0,0,0,0,0,0,0,0,0,0,0,0,0,0,0,0,0,0,0,0,0,0,0,0,0,0,0,0,0,0,0,0},

{0,0,0,0,0,0,0,0,0,0,0,0,0,0,0,0,0,0,0,0,0,0,0,0,0,0,0,0,0,0,0,0,0,0,0,0,0,0,0,0,0,0,0,0,0,0,0,0,0,0,0,0,0,0,0,0,0,0,0,0,0,0,0,0,0,0,0,0,0,0,0,0,0,0,0,0,0,0,0,0,0,0,0,0,0,0,0,0,0,0,0,0,0,0,0,0,0,0,0,0},

{0,0,0,0,0,0,0,0,0,0,0,0,0,0,0,0,0,0,0,0,0,0,0,0,0,0,0,0,0,0,0,0,0,0,0,0,0,0,0,0,0,0,0,0,0,0,0,0,0,0,0,0,0,0,0,0,0,0,0,0,0,0,0,0,0,0,0,0,0,0,0,0,0,0,0,0,0,0,0,0,0,0,0,0,0,0,0,0,0,0,0,0,0,0,0,0,0,0,0,0},

{0,0,0,0,0,0,0,0,0,0,0,0,0,0,0,0,0,0,0,0,0,0,0,0,0,0,0,0,0,0,0,0,0,0,0,0,0,0,0,0,0,0,0,0,0,0,0,0,0,0,0,0,0,0,0,0,0,0,0,0,0,0,0,0,0,0,0,0,0,0,0,0,0,0,0,0,0,0,0,0,0,0,0,0,0,0,0,0,0,0,0,0,0,0,0,0,0,0,0,0},

{0,0,0,0,0,0,0,0,0,0,0,0,0,0,0,0,0,0,0,0,0,0,0,0,0,0,0,0,0,0,0,0,0,0,0,0,0,0,0,0,0,0,0,0,0,0,0,0,0,0,0,0,0,0,0,0,0,0,0,0,0,0,0,0,0,0,0,0,0,0,0,0,0,0,0,0,0,0,0,0,0,0,0,0,0,0,0,0,0,0,0,0,0,0,0,0,0,0,0,0},

{0,0,0,0,0,0,0,0,0,0,0,0,0,0,0,0,0,0,0,0,0,0,0,0,0,0,0,0,0,0,0,0,0,0,0,0,0,0,0,0,0,0,0,0,0,0,0,0,0,0,0,0,0,0,0,0,0,0,0,0,0,0,0,0,0,0,0,0,0,0,0,0,0,0,0,0,0,0,0,0,0,0,0,0,0,0,0,0,0,0,0,0,0,0,0,0,0,0,0,0}

}

Referenced by Landscape::SupplyWindDirection().


- CJT
- MSVC
- ALMaSS Working Source
- Landscape
- landscape.h
- Generated on Thu Jan 10 2013 13:15:35 for ALMaSS Skylark ODdox by
   1.8.1.1
